# Pertuzumab plus high-dose trastuzumab for HER2-positive breast cancer with brain metastases: PATRICIA final efficacy data

**DOI:** 10.1038/s41523-023-00587-2

**Published:** 2023-11-17

**Authors:** Nancy U. Lin, Priya Kumthekar, Solmaz Sahebjam, Nuhad Ibrahim, Anita Fung, Anna Cheng, Alan Nicholas, Jesse Sussell, Mark Pegram

**Affiliations:** 1 Dana-Farber Cancer Institute, Boston, MA USA; 2 Northwestern University, Feinberg School of Medicine, Chicago, IL USA; 3 Moffitt Cancer Center, University of South Florida, Tampa, FL USA; 4 MD Anderson Cancer Center, Houston, TX USA; 5 Genentech Inc., South San Francisco, CA USA; 6 Stanford Comprehensive Cancer Institute, Palo Alto, CA USA; 7 Present Address: Department of Oncology, Johns Hopkins School of Medicine, Baltimore, MD USA

**Keywords:** Breast cancer, Breast cancer

## Abstract

The PATRICIA study (NCT02536339) examined the efficacy and safety of pertuzumab plus high-dose trastuzumab in patients with HER2-positive metastatic breast cancer (MBC) with progressive central nervous system (CNS) metastases following radiotherapy. Primary analysis confirmed CNS objective response rate (ORR) was 11% (95% confidence interval [CI]: 3–25); clinical benefit rate (CBR) was 68% (4 months) and 51% (6 months). We report final efficacy data after a further 21-months of follow-up, updated safety, survival, and patient-reported outcomes (PROs). Patients received standard-dose pertuzumab plus high-dose trastuzumab (6 mg/kg weekly) until CNS or systemic disease progression or unacceptable toxicity. Primary endpoint: confirmed ORR (CNS) per Response Assessment in Neuro-Oncology Brain Metastases criteria. Secondary endpoints were response duration, CBR, progression-free survival (PFS), overall survival (OS), safety, and PROs. By clinical cut-off, 39 patients had completed or discontinued treatment. Confirmed ORR (CNS) was 11% (95% CI: 3.0–25.4). Median CNS-PFS was 4.6 months (95% CI: 4.0–8.9), as was median CNS-PFS or systemic PFS (95% CI: 4.0–8.9); median OS was 27.2 months (95% CI: 16.1–not reached). CBR in the CNS was 51% (19 patients, 95% CI: 34.4–68.1) at 6 months. Two patients remained on treatment until study closure, achieving stable disease for 4.1 and 4.8 years. Treatment-related grade 3/4 adverse events occurred in 7.7% of patients. Patients with confirmed partial response or stable disease (≥4 months) in the CNS had stable PROs over time. Pertuzumab plus high-dose trastuzumab represents a reasonable non-chemotherapeutic treatment option for selected patients with HER2-positive MBC with CNS metastases.

## Introduction

The development of brain metastases is estimated to occur in 40–50% of patients with HER2-positive or triple-negative metastatic breast cancer (MBC)^1–3^. Patients with HER2-positive MBC and central nervous system (CNS) metastases generally experience shorter survival and report poorer quality of life (QoL) than those without CNS metastases, including more severe cognitive dysfunction and symptom interference^4^.

Recording symptoms and functional impacts of treatment from the patients’ perspective within a clinical trial setting provides added value to the standard outcome measures. In this regard, the United States Food and Drug Administration (FDA) recently issued guidance on the importance of collecting core patient-reported outcomes (PROs), particularly in trials of anti-cancer therapies^5^. The standard of care for patients presenting with brain metastases secondary to HER2-positive MBC has traditionally been local therapy, using surgery and/or radiotherapy, with systemic therapy added for extracranial disease control. In some cases, systemic therapy is prescribed for the purpose of intracranial disease control. However, upon commencement of the Phase II PATRICIA study (NCT02536339) there were no FDA-approved systemic therapies for this indication.

The PATRICIA study examined the efficacy and safety of standard-dose intravenous (i.v.) pertuzumab in combination with i.v. high-dose trastuzumab (6 mg/kg weekly) in patients with HER2-positive MBC with CNS metastases that had progressed following radiotherapy. The hypothesis, based on preclinical data^6^, was that high-dose trastuzumab would be efficacious in the CNS for patients who had progressed on standard-dose trastuzumab without causing further cardiac toxicity. At the primary analysis of the PATRICIA study (median [min–max] follow-up 16.6 months [0.8–37.5]), confirmed objective response rate (ORR) in the CNS was 11% (95% confidence interval [CI]: 3–25), with a clinical benefit rate (CBR) of 68% and 51% at 4 and 6 months, respectively. No new safety signals were observed for either pertuzumab or high-dose trastuzumab^1^. Of note, based upon the PATRICIA clinical data, the NCCN Clinical Practice guidelines in Oncology (NCCN Guidelines^®^) for the management of CNS cancers were first updated in June 2022 to include pertuzumab and high-dose trastuzumab as a new category 2A regimen for patients with HER2-positive MBC and brain metastases (Referenced with permission from the NCCN Clinical Practice Guidelines in Oncology (NCCN Guidelines®) for Central Nervous System Cancers V.1.2023. © National Comprehensive Cancer Network, Inc. 2023. All rights reserved. Accessed [June 01, 2023]. To view the most recent and complete version of the guideline, go online to NCCN.org. NCCN makes no warranties of any kind whatsoever regarding their content, use or application and disclaims any responsibility for their application or use in any way.).

Here, we present the final efficacy analysis of the PATRICIA study after a further 21 months of follow-up, as well as updated safety data, survival, and PROs.

## Results

### Patients

Between December 15, 2015 and May 18, 2017, 40 patients were enrolled across 16 sites. One patient did not receive study treatment, therefore the safety population comprised 39 patients. The efficacy-evaluable population included 37 patients: one patient did not receive study treatment and two patients had no post-baseline assessments due to withdrawal (*n* = 1) and treatment discontinuation as a result of symptomatic deterioration (*n* = 1).

**Fig. 1:**
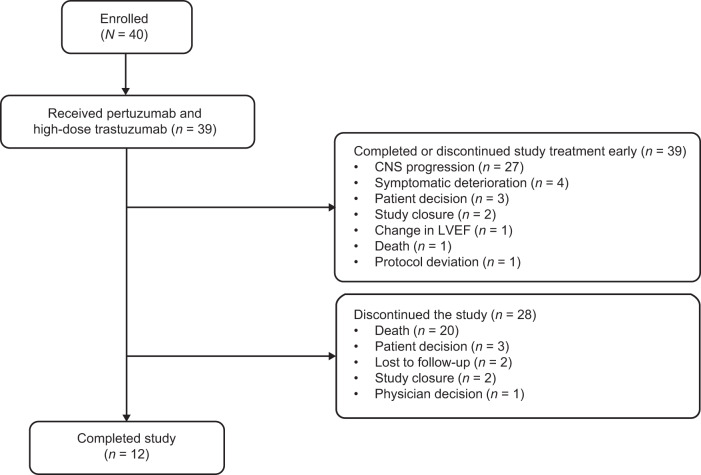
Patient flow (CONSORT) diagram. CNS central nervous system, LVEF left ventricular ejection fraction.

By the clinical cut-off date (February 10, 2021), all 39 patients had completed or discontinued study treatment, 17 patients (43.6%) had discontinued pertuzumab, and one patient (2.6%) had discontinued both pertuzumab and trastuzumab. The primary reason for treatment discontinuation was CNS progression (*n* = 27/39 [69.2%]; Fig. 1). Two patients remained on study treatment until the study closed and achieved stable disease for 4.1 and 4.8 years. All patients completed (*n* = 12) or discontinued (*n* = 28) the study, with cancer mortality being the most common cause of study discontinuation (*n* = 20/28 [71.4%]; Fig. 1).

Patients had a median age of 48 years (range 34–69) and were primarily White (*n* = 36 [90.0%]; Table 1). At baseline, extracranial disease was present in 24 patients (60.0%) and 11 patients (27.5%) were receiving concomitant systemic treatment for MBC. Median time from brain metastasis diagnosis until study entry was 19.4 months (range 3.1–65.5). All patients had received prior radiotherapy. Median time from last CNS-directed radiotherapy was 18.6 months (range 2.8–63.1). Most patients (*n* = 28/40 [70.0%]) had received prior whole-brain radiotherapy (WBRT) at first diagnosis of CNS metastases, and stereotactic radiosurgery (SRS) at the time of CNS progression (*n* = 18/40 [54.5%]) (Supplementary Table 1).

**Table 1:** Baseline demographic and disease characteristics^a^.

Characteristic	ITT population (*N* = 40)
Median age, years (range)	48 (34–69)
Females, *n* (%)	40 (100.0)
Race, *n* (%)	
White	36 (90.0)
Asian	2 (5.0)
Other	1 (2.5)
Not reported	1 (2.5)
ECOG PS, *n* (%)	*n* = 39
0	13 (33.3)
1	26 (66.7)
Median LVEF, % (range)	*n* = 39
	60 (50–75)
Breast cancer stage at initial diagnosis, *n* (%)	
0	1 (2.5)
I	2 (5.0)
II	9 (22.5)
III	10 (25.0)
IV	18 (45.0)
Patients with extracranial disease, *n* (%)	24 (60.0)
Measurable	8 (20.0)
Non-measurable	16 (40.0)
Prior CNS radiotherapy, *n* (%)	*n* = 39
WBRT only	16 (41.0)
SRS only	11 (28.2)
Both WBRT and SRS	12 (30.8)
Prior craniotomy, *n* (%)	6 (15.0)
Prior HER2-targeted treatment for MBC, *n* (%)^a^	
Trastuzumab plus pertuzumab	19 (47.5)
Trastuzumab only	15 (37.5)
T-DM1	15 (37.5)
Lapatinib	18 (45.0)
Neratinib	3 (7.5)
Median number of prior chemotherapy agents, *n* (range)	*n* = 17
	3 (2–5)
Concomitant on-study systemic treatment for MBC, *n* (%)^a,b^	*n* = 11
Anastrozole	1 (9.1)
Capecitabine	3 (27.3)
Exemestane	1 (9.1)
Fulvestrant	1 (9.1)
Gemcitabine	1 (9.1)
Letrozole	2 (18.2)
Palbociclib	3 (27.3)
Vinorelbine	1 (9.1)
Concomitant antiepileptics use during study, *n* (%)^a^	*n* = *29*
Acetazolamide	1 (3.4)
Cannabis	3 (10.3)
Clonazepam	1 (3.4)
Diazepam	2 (6.9)
Gabapentin	9 (31.0)
Lacosamide	3 (10.3)
Levetiracetam	17 (58.6)
Lorazepam	14 (48.3)
Midazolam	1 (3.4)
Topiramate	1 (3.4)
Zonisamide	1 (3.4)
Concomitant corticosteroid use during study, *n* (%)	
No	29 (72.5)
Yes	11 (27.5)
Dexamethasone^a,b^	11 (100.0)
Other^a,b^	1 (9.1)
Median time from onset of brain metastases to first dose of study treatment, months (range)	19.4 (3.1–65.5)

*CNS* central nervous system, *ECOG PS* Eastern Cooperative Oncology Group performance status, *HER2* human epidermal growth factor receptor 2, *ITT* intent to treat, *LVEF* left ventricular ejection fraction, *MBC* metastatic breast cancer, *SRS* stereotactic radiosurgery, *T-DM1* trastuzumab emtansine, *WBRT* whole-brain radiotherapy.

^a^Patients may have received multiple treatments.

^b^Calculated based on *n*, the number of patients with at least one treatment.

### Clinical outcomes

**Fig. 2:**
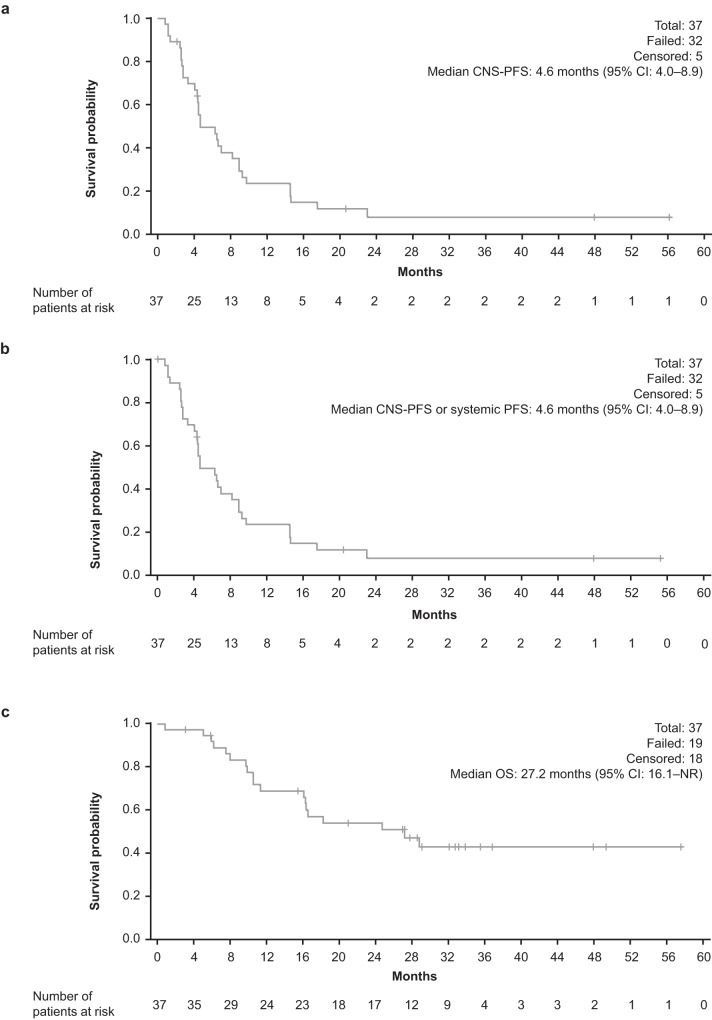
Kaplan–Meier survival plots. **a** CNS-PFS; **b** CNS-PFS or systemic PFS; **c** OS. CI confidence interval, CNS central nervous system, NR not reached, OS overall survival, PFS progression-free survival.

ORR within the CNS per Response Assessment in Neuro-Oncology Brain Metastases (RANO-BM) criteria^7^ was 11% (four patients, 95% CI: 3.0–25.4), with a median duration of response (DOR) of 4.6 months (95% CI: 3.3–5.6). Clinical history and pathological features from each of the four patients who responded are outlined in Supplementary Table 2, with examples of clinical images in Supplementary Fig. 1. CBR in the CNS was 68% (25 patients, 95% CI: 50.2–82.0) at 4 months, and 51% (19 patients, 95% CI: 34.4–68.1) at 6 months^1^. Median DOR for the four patients was the same as that in the primary analysis despite additional follow-up. Median systemic progression-free survival (PFS) was 16.3 months (95% CI: 9.9–24.7). Median CNS-PFS was 4.6 months (95% CI: 4.0–8.9) (Fig. 2a), and median CNS-PFS or systemic PFS was 4.6 months (95% CI: 4.0–8.9) (Fig. 2b). Median overall survival (OS) was 27.2 months (95% CI: 16.1–not reached) (Fig. 2c). The 1-year CNS-PFS was 20.4% (95% CI: 9.0–35.0) and 1-year OS was 68.7% (95% CI: 50.7–81.3).

CBR for patients who received concomitant antiepileptics vs. those who did not was 62.1% vs. 87.5% at 4 months, and 51.7% vs. 50.0% at 6 months, respectively. CBR for patients who received concomitant MBC treatment vs. those who did not was 90.0% vs. 59.3% at 4 months, and 80.0% vs. 40.7% at 6 months, respectively. CBR for patients who received corticosteroids vs. those who did not was 63.6% vs. 69.2% at 4 months, and 45.5% vs. 53.8% at 6 months, respectively. CBR with a positive primary tumor hormone receptor status vs. negative status was 68.4% vs. 66.7% at 4 months, and 57.9% vs. 44.4% at 6 months, respectively (Table 2).

**Table 2:** Clinical benefit rate at 4 and 6 months in patient subgroups within the efficacy-evaluable population.

	Efficacy-evaluable population (*N* = 37)	
	Received concomitant antiepileptics (*n* = 29)	Did not receive concomitant antiepileptics (*n* = 8)
CBR at 4 months, *n* (%)	18/29 (62.1)	7/8 (87.5)
CBR at 6 months, *n* (%)	15/29 (51.7)	4/8 (50.0)
	Received concomitant systemic MBC treatment (*n* = 10)	Did not receive concomitant systemic MBC treatment (*n* = 27)
CBR at 4 months, *n* (%)	9/10 (90.0)	16/27 (59.3)
CBR at 6 months, *n* (%)	8/10 (80.0)	11/27 (40.7)
	Received corticosteroids (*n* = 11)	Did not receive corticosteroids (*n* = 26)
CBR at 4 months, *n* (%)	7/11 (63.6)	18/26 (69.2)
CBR at 6 months, *n* (%)	5/11 (45.5)	14/26 (53.8)
	Hormone receptor-positive primary tumor (*n* = 19)	Hormone receptor-negative primary tumor (*n* = 18)
CBR at 4 months, *n* (%)	13/19 (68.4)	12/18 (66.7)
CBR at 6 months, *n* (%)	11/19 (57.9)	8/18 (44.4)
	Baseline ECOG PS 0 (*n* = 13)	Baseline ECOG PS 1 (*n* = 24)
CBR at 4 months, *n* (%)	11/13 (84.6)	14/24 (58.3)
CBR at 6 months, *n* (%)	9/13 (69.2)	10/24 (41.7)

*CBR* clinical benefit rate, *ECOG PS* Eastern Cooperative Oncology Group performance status, *MBC* metastatic breast cancer.

### Safety

Median treatment duration was 21.0 weeks (range 3.0–249.9) with pertuzumab and 20.3 weeks (range 2.0–249.9) with trastuzumab. Patients received a median of 7 cycles (range 1–76) of pertuzumab and 20 cycles (range 2–222) of trastuzumab. Overall, 38 patients (97.4%) experienced treatment-emergent adverse events (AEs), which were primarily grade 1/2 in severity (53.8; Table 3). Seventeen patients (43.6%) had grade 3/4 AEs; there were no grade 5 AEs. The most frequent AEs were diarrhea (23 patients, 59.0%), fatigue (17 patients, 43.6%), and nausea and vomiting (12 patients each, 30.8%).

**Table 3:** Treatment-emergent AEs reported in ≥10% of patients.

Preferred term, *n* (%)	Safety population (*N* = 39)	NCI CTCAE			
		Grade 1	Grade 2	Grade 3	Grade 4
Patients with ≥1 AE	38 (97.4)	5 (12.8)	16 (41.0)	14 (35.9)	3 (7.7)
Diarrhea	23 (59.0)	17 (43.6)	6 (15.4)	0	0
Fatigue	17 (43.6)	7 (17.9)	8 (20.5)	2 (5.1)	0
Nausea	12 (30.8)	11 (28.2)	1 (2.6)	0	0
Vomiting	12 (30.8)	9 (23.1)	3 (7.7)	0	0
Constipation	7 (17.9)	6 (15.4)	1 (2.6)	0	0
Dizziness	7 (17.9)	3 (7.7)	4 (10.3)	0	0
Headache	7 (17.9)	2 (5.1)	4 (10.3)	1 (2.6)	0
Insomnia	7 (17.9)	5 (12.8)	2 (5.1)	0	0
Asthenia	6 (15.4)	2 (5.1)	3 (7.7)	1 (2.6)	0
Decreased appetite	6 (15.4)	5 (12.8)	1 (2.6)	0	0
Hypokalemia	6 (15.4)	5 (12.8)	0	1 (2.6)	0
Rash	6 (15.4)	6 (15.4)	0	0	0
Seizure	6 (15.4)	2 (5.1)	0	3 (7.7)	1 (2.6)
Upper respiratory tract infection	6 (15.4)	3 (7.7)	3 (7.7)	0	0
Anxiety	5 (12.8)	4 (10.3)	1 (2.6)	0	0
Cough	5 (12.8)	4 (10.3)	1 (2.6)	0	0
Gait disturbance	5 (12.8)	2 (5.1)	3 (7.7)	0	0
Nasal congestion	5 (12.8)	4 (10.3)	1 (2.6)	0	0
Pruritus	5 (12.8)	5 (12.8)	0	0	0
Urinary tract infection	5 (12.8)	0	4 (10.3)	1 (2.6)	0
Arthralgia	4 (10.3)	2 (5.1)	2 (5.1)	0	0
Back pain	4 (10.3)	2 (5.1)	2 (5.1)	0	0
Dysphagia	4 (10.3)	3 (7.7)	1 (2.6)	0	0
Fall	4 (10.3)	2 (5.1)	2 (5.1)	0	0
Hypertension	4 (10.3)	0	2 (5.1)	1 (2.6)	1 (2.6)
Muscle spasms	4 (10.3)	4 (10.3)	0	0	0
Pain	4 (10.3)	3 (7.7)	1 (2.6)	0	0
Pain in extremity	4 (10.3)	3 (7.7)	1 (2.6)	0	0
Paresthesia	4 (10.3)	4 (10.3)	0	0	0
Vision blurred	4 (10.3)	2 (5.1)	2 (5.1)	0	0

*AEs* adverse events, *NCI CTCAE* National Cancer Institute Common Terminology Criteria for Adverse Events.

Treatment-related AEs occurred in 30 patients (76.9%), most commonly diarrhea (16 patients, 41.0%) and fatigue (11 patients, 28.2%), and the majority of these (66.7%) were grade 1/2 in severity. Three patients (7.7%) experienced treatment-related grade 3/4 AEs, including grade 3 left ventricular dysfunction, asthenia, and fatigue, and grade 4 hypertension. Serious AEs occurred in seven patients (17.9%) (Table 4). Of these, five patients (12.8%) had at least one grade 3 serious AE of seizure, hydrocephalus, viral gastroenteritis, and parainfluenza virus infection. One patient (2.6%) had a serious AE of grade 4 hypertension that was considered related to study treatment. No grade 5 serious AEs were reported.

**Table 4:** Serious treatment-emergent AEs.

Preferred term, *n* (%)	Safety population (*N* = 39)	NCI CTCAE			
		Grade 1	Grade 2	Grade 3	Grade 4
Patients with ≥1 serious AE	7 (17.9)	0	1 (2.6)	5 (12.8)	2 (5.1)
Gastroenteritis viral	1 (2.6)	0	0	1 (2.6)	0
Parainfluenza virus infection	1 (2.6)	0	0	1 (2.6)	0
Seizure	4 (10.3)	0	0	3 (7.7)	1 (2.6)
Headache	1 (2.6)	0	1 (2.6)	0	0
Hydrocephalus	1 (2.6)	0	0	1 (2.6)	0
Hypertension	1 (2.6)	0	0	0	1 (2.6)

Includes all serious AEs occurring on or after Day 1 of study treatment until 30 days after the last dose of study treatment. Multiple occurrences of AEs for a patient were counted only once at the highest NCI CTCAE grade.

*AEs* adverse events, *NCI CTCAE* National Cancer Institute Common Terminology Criteria for Adverse Events.

Two patients discontinued both pertuzumab and trastuzumab due to AEs: one patient with prior cardiac history experienced grade 3 left ventricular dysfunction that was considered related to treatment, and another patient had grade 3 seizure (reported as a serious AE) that was considered not related to treatment.

No clinically meaningful changes in mean or median left ventricular ejection fraction (LVEF) levels were observed over time in an exploratory post-hoc analysis using only study visits with ≥50% non-missing LVEF data (screening, Week 6, and Week 12)^8^. No new safety signals, including cardiac safety signals, were observed.

### Patient-reported outcomes

Among the 36 patients (90.0%) in the PRO population, mean MD Anderson Symptom Inventory-Brain Tumor (MDASI-BT) module^9^ symptom severity and symptom interference scores were generally stable over time. Mean (standard deviation; SD) symptom severity scores were 1.7 (1.6) at baseline, 2.2 (2.1) at Week 12, and 1.9 (2.5) at Week 28. Mean (SD) symptom interference scores at these time points were 2.5 (2.6), 2.8 (3.4), and 1.8 (2.4), respectively. Mean (SD) change from baseline to Week 12 in symptom severity and interference scores was 0.3 (1.5) and 0.3 (3.1), respectively.

**Fig. 3:**
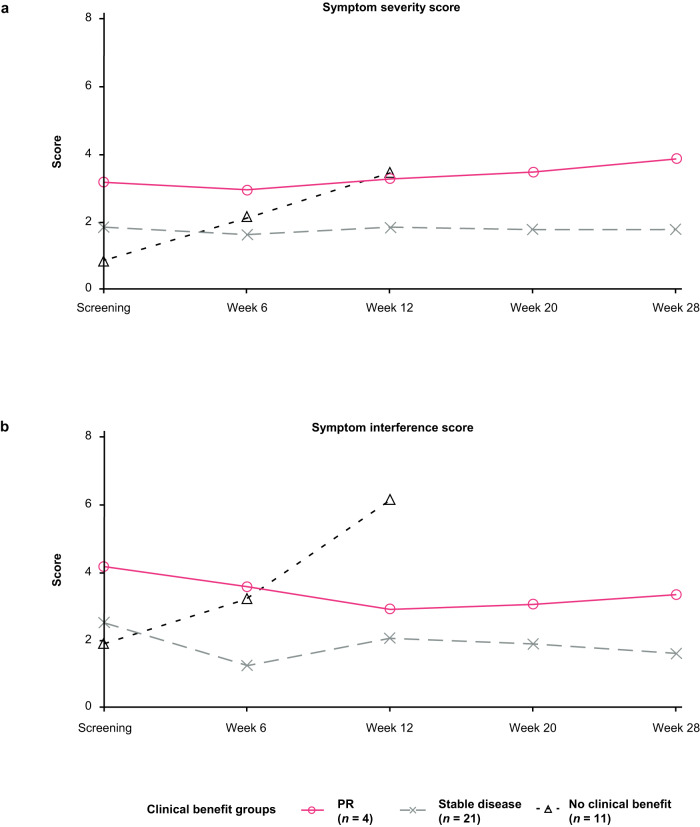
Mean MDASI-BT scale scores over time by clinical benefit group. **a** symptom severity scores; **b** symptom interference scores. MDASI-BT MD Anderson Symptom Inventory-Brain Tumor, PR partial response.

On average, patients who achieved confirmed partial response (PR) or who had stable disease (≥4 months) in the CNS following treatment showed stable symptom severity and symptom interference scores over time, while scores worsened over time in patients without clinical benefit (Fig. 3). Sensitivity analyses restricted to patients with non-missing MDASI-BT symptom severity or symptom interference scores at Week 12 showed similar results to the overall PRO population. Patients with a baseline Eastern Cooperative Oncology Group performance status (ECOG PS) of 0 had similar mean MDASI-BT symptom severity scores at Week 12, but those with a baseline ECOG PS of 1 generally had worse scores over time (Supplementary Table 3). Patients with a highest AE grade of 0–2 during the study had consistently better mean MDASI-BT symptom and interference scores than those with a highest AE grade of 3–4 (Supplementary Fig. 2a). No difference in mean MDASI-BT symptom or interference scores was noted in patients with a highest treatment-related AE grade of 0–1 or 2–4 (Supplementary Fig. 2b).

CBR in the CNS at 6 months was higher in patients with baseline MDASI-BT scores above the median vs. at or below the median, for both symptom sever (65% vs. 42%, respectively) and symptom interference (61% vs. 44%, respectively; Supplementary Table 4).

## Discussion

In this final efficacy analysis of the PATRICIA study in patients with HER2-positive MBC with brain metastases that had progressed following radiotherapy, median CNS-PFS was 4.6 months, median CNS-PFS or systemic PFS was also 4.6 months, and median OS was 27.2 months. CBR in the CNS was 68% (95% CI: 50.2–82.0) at 4 months and 51% (95% CI: 34.4–68.1) at 6 months^1^. Remarkably, two patients experienced stable disease both intracranially and extracranially for 4.1 and 4.8 years, respectively. The median OS of 27.2 months is also notable in this heavily pre-treated population of patients who had all progressed after WBRT and/or SRS treatment prior to study entry. As median time from last radiotherapy to study entry was 18.6 months (range 2.8–63.1), we believe findings from the study can be firmly attributed to the systemic regimen.

At the time of study initiation, no approved systemic therapies existed for HER2-positive MBC with brain metastases. In 2020, the FDA approved tucatinib in combination with trastuzumab and capecitabine for the treatment of patients with HER2-positive MBC who have previously received anti-HER2-based therapy, including those with brain metastases. The approval was based on results of the randomized Phase II HER2CLIMB study (NCT02614794), which reported superior PFS with tucatinib vs. placebo both when given in combination with trastuzumab and capecitabine, in the overall study population and in patients with brain metastases at baseline (PFS hazard ratio [HR] 0.48; 95% CI: 0.34–0.69; *p* < 0.001)^10^. Remarkably, this is the first FDA approval that specifies patients with brain metastases in the indication statement^11^. However, most patients progress while receiving tucatinib combinations, and it is not known whether there is a role for continued tucatinib or other HER2-targeted tyrosine kinase inhibitors (TKIs) in subsequent lines of therapy following progression^12^. This highlights an unmet medical need for multiple sequential CNS-active therapies in this setting.

While being cautious regarding cross-trial comparisons, the HER2CLIMB study (NCT02614794) reported 1-year PFS in patients with brain metastases of 24.9% in the tucatinib-combination group with median PFS of 7.6 months, as well as an OS advantage compared with the trastuzumab-capecitabine control arm. As such, we believe that the tucatinib, trastuzumab, and capecitabine regimen should generally be sequenced prior to the PATRICIA regimen. Further studies could investigate whether prior HER2 TKI exposure influences the efficacy of pertuzumab plus high-dose trastuzumab in patients with active brain metastases.

A number of study groups have reported activity against CNS metastases with HER2-targeted monoclonal antibodies and antibody-drug conjugates (ADCs). Addition of pertuzumab to first-line trastuzumab and docetaxel in the Phase III CLEOPATRA study (NCT00567190) significantly prolonged PFS (HR 0.62, 95% CI: 0.51–0.75; *p* < 0.001) compared with placebo with trastuzumab and docetaxel^13^, and also delayed the time to onset of CNS disease (HR 0.58, 95% CI: 0.39–0.85; *p* = 0.0049)^14^. Also, in the Phase IIIb KAMILLA study, trastuzumab emtansine (T-DM1) treatment resulted in regression of brain metastases in patients with HER2-positive breast cancer^15^. In the randomized, Phase III DESTINY-Breast03 head-to-head study (NCT03529110), intracranial ORR was 63.9% with T-DXd compared with 33.4% with T-DM1 among patients with stable brain metastases at baseline^16^. Finally, in the prospective, open-label, single-arm, Phase II TUXEDO-1 trial, of 15 patients enrolled in the intent-to-treat population who received at least one dose of trastuzumab deruxtecan (T-DXd), overall intracranial response rate was 73.3% (95% CI: 48.1–89.1)^17^. Taken together with our data, these findings suggest that both monoclonal antibodies and ADCs have CNS activity.

Notably, neither trastuzumab nor pertuzumab are thought to penetrate the intact blood–brain barrier (BBB)^18^. However, monoclonal antibodies do appear to cross the disrupted blood–tumor barrier (BTB) within CNS metastatic tumor microenvironments^6^. According to Le Chatelier’s principle, which states that changes in the temperature, pressure, volume, or in the present case, concentration of a system will result in predictable and opposing changes in the system to achieve a new equilibrium state^19^, our results suggest that increased systemic trastuzumab exposure may result in CNS efficacy, possibly by driving higher concentrations of trastuzumab into brain metastases. While the BTB is generally more permeable than the intact BBB, techniques to disrupt the BBB/BTB and enhance delivery of drugs to brain tumors, such as focused transcranial ultrasound with intravenously delivered microbubbles^20^, high-affinity antibodies that exploit endogenous receptor-mediated transport systems^21,22^, and nanoparticle-mediated delivery of macromolecules^23^, may provide additional benefits. Furthermore, there is evidence that BBB/BTB integrity is altered after the application of ionizing radiotherapy^24,25^. Notably, all patients in the present study had received prior radiation therapy to the brain, which may have facilitated greater tumor penetration of the macromolecular therapeutic antibodies used, including pertuzumab and high-dose trastuzumab, in particular.

Importantly, with additional follow-up, pertuzumab plus high-dose trastuzumab did not lead to the emergence of any new safety signals or to further cardiotoxicity relative to the primary analysis of PATRICIA. With caution related to cross-trial comparisons, it appears that the addition of high-dose trastuzumab to pertuzumab in our study was also associated with a more favorable toxicity profile than other combination regimens investigated in randomized clinical trials in HER2-positive MBC. In PATRICIA, treatment-related grade 3/4 AEs were reported in only 7.7% of patients, with 5.1% of patients discontinuing due to AEs. By contrast, in the HER2CLIMB study of tucatinib or placebo in combination with trastuzumab and capecitabine, grade ≥3 treatment-emergent AEs were observed in 60.6% and 51.3% of patients, respectively^12^. Additionally, treatment-emergent AEs led to the discontinuation of tucatinib and capecitabine in 5.9% and 11.6% of patients, respectively^12^. Treatment-related grade 3/4 AEs were reported in 45.1% of patients receiving T-DXd and 39.8% of patients receiving T-DM1 in the DESTINY-Breast03 study^26^. In total, 13.6% and 7.3% of patients discontinued T-DXd and T-DM1, respectively, due to AEs.

In general, patients treated with pertuzumab plus high-dose trastuzumab who had a PR or stable disease in the CNS, had stable PROs for symptom severity and symptom interference with daily life over 12 weeks, while patients without clinical benefit from treatment reported worsened outcomes. Worsened PROs were also associated with the highest grade AEs, providing evidence of the validity of the PRO tool that was selected for use in this study population. Thus, the PRO data from PATRICIA support the idea that the CBR measured in the study directly translated into a meaningful improvement in patient QoL. FDA guidance recommends that consideration should be given to the inclusion of patients with brain metastases in cancer clinical trials^27^; this recommendation ensures that therapies intended to treat brain metastases have a wider impact for patients, e.g., in terms of QoL improvement. The low toxicity observed in the PATRICIA study is also consistent with a lack of detrimental impact of treatment on patient-reported QoL.

The study was limited by its relatively small sample size. In addition, as eligible patients were required to have had prior radiotherapy for CNS metastases, documented disease progression (PD) in the CNS, and stable extracranial disease, PATRICIA represents only a subset of the HER2-positive MBC patient population with brain metastases. Newer systemic interventions, including those that improve OS, have become available since the PATRICIA study was conducted^10,16^. Lastly, PRO analyses in oncology are subject to differential missingness, whereby follow-up assessments are not always possible, particularly in patients with limited or no clinical benefit, e.g., those with PD or who have died. In this study, the small sample sizes combined with missingness of PRO data precluded the use of formal statistical tests to assess PROs.

Multi-time-point PROs are not always collected in oncology clinical trials, yet PATRICIA demonstrated the feasibility and importance of these endpoints. Future trials should include methodical PRO assessments at defined time points, including the period following treatment discontinuation to fully capture the trajectory of a patient’s QoL during and after treatment. New technologies to gather PRO data (e.g., wearable devices) are also being investigated and may be integrated into future clinical trials^28^.

In summary, this single-arm, multicenter, Phase II study in patients with active HER2-positive breast cancer brain metastases (defined as CNS PD following prior radiation therapy), demonstrated that high-dose trastuzumab (6 mg/kg i.v. every week) combined with 3-weekly pertuzumab (given in standard i.v. dose and schedule) was associated with a high rate of clinical benefit, including radiographic stability and maintenance of QoL. Based on these findings, the National Comprehensive Cancer Network® (NCCN®) recently added pertuzumab plus high-dose trastuzumab as a category 2A regimen for patients with HER2-positive MBC and brain metastases (Referenced with permission from the NCCN Clinical Practice Guidelines in Oncology (NCCN Guidelines®) for Central Nervous System Cancers V.1.2023. © National Comprehensive Cancer Network, Inc. 2023. All rights reserved. Accessed [June 01, 2023]. To view the most recent and complete version of the guideline, go online to NCCN.org. NCCN makes no warranties of any kind whatsoever regarding their content, use or application and disclaims any responsibility for their application or use in any way.). Although the current study was small, in light of the high clinical need and limited number of available effective regimens, pertuzumab plus high-dose trastuzumab may represent a reasonable treatment option for selected patients with HER2-positive MBC with CNS metastases, given its high therapeutic index (benefit/toxicity profile), particularly the high CBR at ≥4 and ≥6 months, and extended survival, however anecdotally, in some patients. Additional research may incorporate biomarker analysis to identify the most suitable patients for this treatment.

## Methods

### Study design and participants

PATRICIA was an open-label, single-arm study conducted in the USA. Full details of the study design have been published previously^1^ and the protocol is available at: https://www.clinicaltrials.gov/ProvidedDocs/39/NCT02536339/Prot_000.pdf. In brief, patients received i.v. pertuzumab (840 mg loading dose, then 420 mg once every 3-week cycle) plus i.v. high-dose trastuzumab (6 mg/kg weekly) until CNS or systemic PD, unacceptable toxicity, withdrawal, or study termination (Supplementary Fig. 3). No dose reductions of study drugs were permitted, and ongoing systemic treatment was to be continued until PD, unacceptable toxicity, study withdrawal, or study closure. Concurrent HER2 TKIs or T-DM1 were not permitted.

Patients aged ≥18 years with confirmed HER2-positive MBC presenting with documented progression in the CNS despite previous radiotherapy, and stable extracranial disease were eligible. Patients were required to have an ECOG PS of 0 or 1, at least one measurable CNS metastasis (≥10 mm per RANO-BM criteria)^7^, and LVEF ≥ 50%. Patients with leptomeningeal disease, symptomatic pulmonary disease, history of intolerance (grade ≥3) or hypersensitivity to study treatment were excluded.

The study protocol was approved by the institutional review board (IRB) or ethics committee at each of the following 16 participating centers: University of Arizona Cancer Center (Western IRB), City of Hope National Medical Center (City of Hope IRB), Stanford Cancer Institute (Research Compliance Office, Stanford University), University of Miami Hospital & Clinics (Western IRB), H. Lee Moffitt Cancer Center and Research Institute (Chesapeake IRB), Northwestern University (Northwestern University Office for Research IRB Office), University of Maryland Medical Center; Department of Neurology (University of Maryland, Baltimore IRB), Associates in Oncology-Hematology, PC (Maryland Oncology-Hematology IRB), Dana-Farber Cancer Institute (Dana Farber Cancer Institute Office for Human Research Studies), Allina Health, Virginia Piper Cancer Institute (Quorum Review, Inc.), Stony Brook University Medical Center (Stony Brook University IRB Committee on Research Involving Human Subjects), Mid Ohio Oncology Hematology; ZangMeister Center (West) (Mid Ohio IRB), Temple Cancer Center; Oncology (Fox Chase Cancer Center IRB), Methodist Hospital Research Institute (Houston Methodist Research Institute IRB), Huntsman Cancer Institute; University of Utah (University of Utah IRB), Northwest Medical Specialties, PLLC (Copernicus Group IRB). The protocol complied with Good Clinical Practice guidelines, the principles of the Declaration of Helsinki, and local laws. All patients provided written informed consent prior to any study-related procedures.

### Outcomes

The primary efficacy endpoint was ORR in the CNS, defined as the proportion of patients with a confirmed complete response (CR) or PR per RANO-BM criteria^7^. The secondary endpoints of DOR (time from first documented CR or PR to PD/death) and CBR (proportion of patients with confirmed CR, PR, or stable disease for ≥4 and ≥6 months) were assessed in the CNS per RANO-BM criteria.

Additional secondary endpoints included: PFS in the CNS (CNS-PFS; time from first dose of study drug to CNS PD or death from any cause, per RANO-BM criteria); systemic PFS (time from first dose of study drug to systemic PD or death from any cause, per Response Evaluation Criteria in Solid Tumors [RECIST] v1.1^29^); CNS-PFS or systemic PFS (time from first dose of study drug to CNS or systemic PD or death from any cause, per RANO-BM and RECIST v1.1); OS (time from first dose of study drug to death from any cause); safety; and PROs, evaluated using the MDASI-BT module^9^.

### Procedures

Responses were assessed by the investigator based on magnetic resonance imaging (MRI) of the brain at Week 6, 12, 20, 28, and every 12 weeks thereafter, until PD. Extracranial responses were measured using MRI, computed tomography, or positron emission tomography at Week 8 and 16, and every 16 weeks thereafter, until PD, as determined by the investigator using RECIST v1.1. AEs were graded according to the NCI Common Terminology Criteria for Adverse Events v4.0. LVEF was assessed at screening, Week 6 and 12, every 12 weeks during the treatment period, and every 6 months during survival follow-up. PROs were evaluated using MDASI-BT module^9^ to determine symptom severity and symptom interference on daily life. Assessments were made at baseline, every brain MRI visit, every 6 weeks x2, then every 8 weeks x2, then every 12 weeks until PD.

### Statistical analysis

The safety population comprised all patients who received any dose of study drugs. The efficacy-evaluable population included all treated patients with at least one follow-up CNS tumor assessment, or those who died without follow-up tumor assessment within 30 days from the last dose of study drug^1^. The PRO population comprised all treated patients with a baseline and at least one post-baseline PRO assessment.

The 95% Clopper-Pearson exact CIs were calculated for ORR and CBR^1^. DOR was estimated using Kaplan–Meier methodology, with 95% CIs for the median time to event calculated using the Brookmeyer-Crowley method. Patients who did not experience PD or death were censored at the last date they were known to be progression free. AEs were summarized using descriptive statistics in patients with ≥1 AE. PROs were also summarized descriptively for the PRO-evaluable population. The clinical cut-off date for the analyses presented in this manuscript was February 10, 2021; two patients were discontinued as a result of study closure.

### Supplementary information

Supplementary material

## Data Availability

Qualified researchers may request access to individual patient level data through the clinical study data request platform (https://vivli.org/). Further details on Roche’s criteria for eligible studies are available here (https://vivli.org/members/ourmembers/). For further details on Roche’s Global Policy on the Sharing of Clinical Information and how to request access to related clinical study documents, see here (https://www.roche.com/research_and_development/who_we_are_how_we_work/clinical_trials/our_commitment_to_data_sharing.htm).
