# Supplementary material for: Pertuzumab plus high-dose trastuzumab for HER2-positive breast cancer with brain metastases: PATRICIA final efficacy data
Source: NPJ Breast Cancer. 2023 Nov 17;9:94. doi: 10.1038/s41523-023-00587-2 (PMC10656527; doi:10.1038/s41523-023-00587-2)
Supplement: Supplementary file 1 — Supplementary material [file 41523_2023_587_MOESM1_ESM.pdf]

Supplementary material

Supplementary Table 1. Prior CNS Treatments

n (%)	ITT population (*N* = 40)
At initial diagnosis of CNS metastases	
WBRT[a]	28 (70.0)
SRS[b]	7 (17.5)
Both WBRT and SRS[c]	0
Other	5 (12.5)
At diagnosis of CNS progression	*n* = 33
WBRT[a]	1 (3.0)
SRS[b]	18 (54.5)
Both WBRT and SRS[c]	1 (3.0)
Other	20 (60.6)
Patients who ever received WBRT or SRS	*n* = 39
WBRT[a]	28 (71.8)
SRS[b]	23 (59.0)
Both WBRT and SRS[c]	12 (30.8)

[a]Patients who had WBRT as one of their initial or progression treatments and a missing or non-WBRT/non-SRS treatment as the other.

[b]Patients who had SRS as one of their initial or progression treatments and a missing or non-WBRT/non-SRS treatment as the other.

[c]Patients who had at least one WBRT and one SRS treatment.

CNS, central nervous system; ITT, intent to treat; SRS, stereotactic radiosurgery; WBRT, whole-brain radiotherapy.

Supplementary Table 2. Clinical history of the four patients who responded[a]

	Patient 1	Patient 2	Patient 3	Patient 4
**Patient demographics**				
Age	42	55	34	40
Sex	Female	Female	Female	Female
Ethnicity	White	White	White	White
**Diagnosis**				
Initial diagnosis[b]	Stage IV T4N1M1	Stage IV T1N1M1	Stage IIIA T2N0M0	Stage IV T1cN0M1
Duration from initial diagnosis to study start	4 years, 9 months, and 2 weeks	1 year and 6 months	4 years and 2 weeks	2 years, 11 months and 3 weeks
HER status	HER2 positive (3+)	HER2 positive (3+)	HER2 positive	HER2 positive (3+)
Disease sites	Brain/CNS, lung, liver and bone	Brain/CNS and liver	Brain/CNS, lung, liver and bone	Brain, lymph nodes, and liver
**Prior treatments**				
Systemic therapy	Doxorubicin + cyclophosphamide; Paclitaxel + trastuzumab; Capecitabine + lapatinib; Trastuzumab; GRN 1005 (clinical trial);	Docetaxel; Trastuzumab; Pertuzumab; Lapatinib; Capecitabine	Paclitaxel; Doxorubicin; Trastuzumab; Pertuzumab + Vinorelbine + Trastuzumab; Capecitabine; Pertuzumab +	Carboplatin + docetaxel + trastuzumab; Ado-tastuzumab emtansine; Capecitabine + lapatinib

	Ado-trastuzumab emtansine		Vinorelbine + Trastuzumab; Ado-trastuzumab emtansine	
Non-CNS-directed local therapy	Bone radiotherapy	None	Radical mastectomy	Chest wall radiotherapy
CNS-directed local therapy	Whole brain radiotherapy	Whole brain radiotherapy	Whole brain radiotherapy	Whole brain radiotherapy; CNS radiotherapy
**On-study treatments**				
Systemic anti-cancer therapy	None	Capecitabine	None	None
Concomitant cortico-steroids for CNS symptoms	Dexamethasone	Dexamethasone	Dexamethasone	Methylprednisolone Dexamethasone
**Medical history**				
Potential CNS-related baseline conditions of interest	Headache, light-headedness, short-term memory loss	Headache, double vision	Seizures, headache, blurred vision, dizziness	Headache, double vision, blurred vision, memory impairment, ataxia, confusion,

				peripheral sensory neuropathy, dizziness
Surgical history	Cholecystectomy and excisional haemorrhoidectomy	None	None	None
ECOG PS	1	1	0	1
LVEF, %	65	58	55	68
**Changes in the study treatment schedule**				
	**Day 1** – IV pertuzumab (840mg) and IV trastuzumab (6mg/kg)	**Day 1** – IV pertuzumab (840mg) and IV trastuzumab (6mg/kg)	**Day 1** – IV pertuzumab (840mg) and IV trastuzumab (6mg/kg)	**Day 1** – IV pertuzumab (840mg) and IV trastuzumab (6mg/kg)
	**Day 21** – Grade 1 blurred vision, no treatment given	**Day 154** – Discontinued treatment due to protocol violation	**Day 134** – Disease progression, treatment discontinued	**Day 272** – disease progression, treatment discontinued
	**Day 212** – Disease progression, treatment discontinued		**Day 498** – Death due to unknown reason	**Day 490** – Death due to unknown reason
	**Day 312** – Systemic progression			

	**Day 504** – Death due to disease progression, blurred vision unsolved	

[a]Patients have been renumbered to protect their anonymity.

[b]All patients were estrogen and progesterone receptor negative.

CNS, Central nervous system; CT, Chemotherapy; ECOG PS, Eastern Cooperative Oncology Group performance status; HER2, human epidermal growth factor receptor 2; IV, intravenous; LVEF, Left ventricular ejection fraction.

Supplementary Table 3. Mean MDASI-BT Scale Scores Over Time by Baseline ECOG PS

	**Symptom severity**		**Symptom interference**	
**Mean (SD)**	**ECOG PS 0**	**ECOG PS 1**	**ECOG PS 0**	**ECOG PS 1**
	n = 13	*n* = 23	*n* = 13	*n* = 23
Baseline	1.7 (1.8)	1.6 (1.5)	2.5 (3.0)	2.5 (2.5)
	n = 11	*n* = 15	*n* = 10	*n* = 14
Week 12	1.7 (2.1)	2.7 (2.1)	1.6 (2.8)	3.7 (3.6)

ECOG PS, Eastern Cooperative Oncology Group performance status; MDASI-BT, MD Anderson Symptom Inventory-Brain Tumor; SD, standard deviation.

Supplementary Table 4. CBR in the CNS at 6 Months by MDASI-BT Scale Scores

	**≤Median**	**>Median**
**Symptom severity score**	*n* = 19	*n* = 17
Patients with CBR, *n* (%) [95% CI]	8 (42) [20–64]	11 (65) [42–87]
Patients without CBR, *n* (%)	11 (58)	6 (35)
**Symptom interference score**	*n* = 18	*n* = 18
Patients with CBR, *n* (%) [95% CI]	8 (44) [22–67]	11 (61) [39–84]
Patients without CBR, *n* (%)	10 (56)	7 (39)

≤Median: Patients with baseline symptom score ≤ baseline median score of 1.75 (0.95).

>Median: Patients with baseline symptom score > baseline median score 1.75 (0.95).

CBR, clinical benefit rate; CI, confidence interval; CNS, central nervous system; MDASI-BT, MD Anderson Symptom Inventory-Brain Tumor.

Supplementary Fig. 1 PATRICIA Study Design

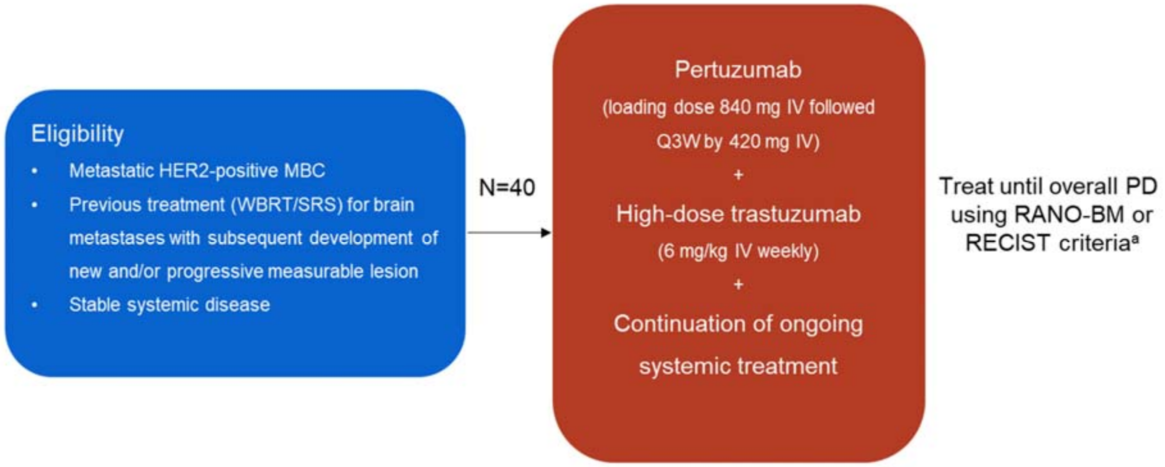

[a]Following a protocol update in October 2018, patients were followed up for 12 months after treatment. Adapted from Lin et al., J Clin Oncol. 2021;39(24):2667-75

HER2, human epidermal growth factor receptor 2; IV, intravenous; MBC, metastatic breast cancer; PD, progressive disease; Q3W, once every 3 weeks; RANO-BM, Response Assessment in Neuro-Oncology Brain Metastases; SRS, stereotactic radiosurgery; WBRT, whole-brain radiotherapy.

Supplementary Fig. 2 Sequential MRI brain scans from a patient on study: (A) Screening; (B) Week 6; (C) Week 12; (D) Week 20.

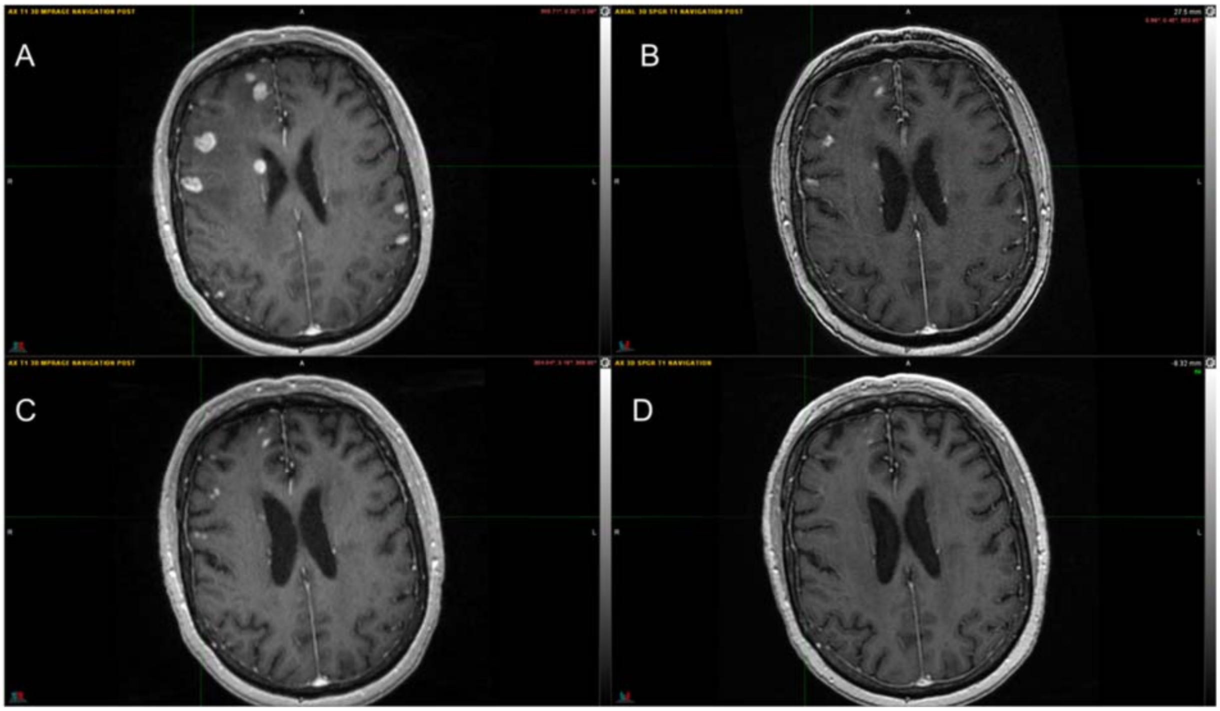

Supplementary Fig. 3 Mean MDASI-BT Scores Over Time by AE Grade: (A) all AEs and (B) Treatment-Related AEs

A

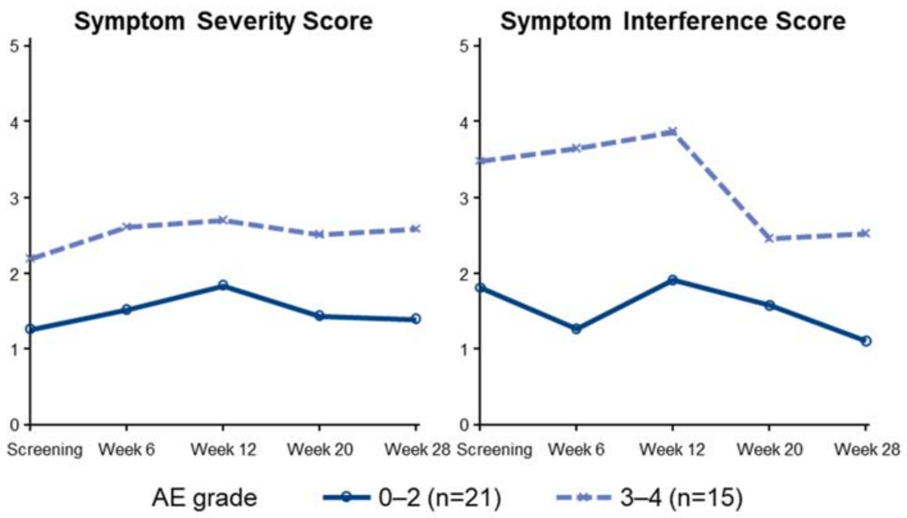

B

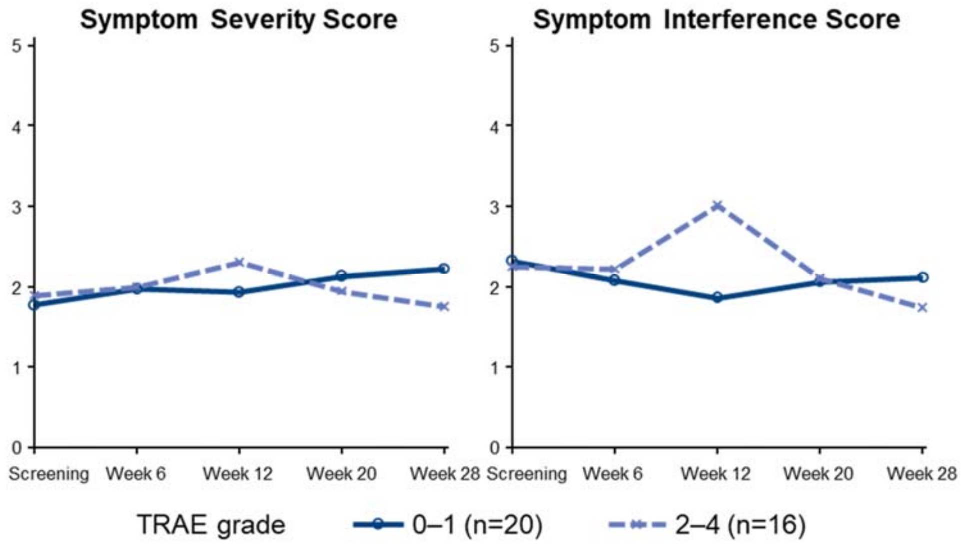

AEs, adverse events; MDASI-BT, MD Anderson Symptom Inventory-Brain Tumor; TRAE, treatment-related adverse events.
